# A Simple and Sensitive Method for Measuring Tumor-Specific T Cell Cytotoxicity

**DOI:** 10.1371/journal.pone.0011867

**Published:** 2010-07-29

**Authors:** Xinping Fu, Lihua Tao, Armando Rivera, Shana Williamson, Xiao-Tong Song, Nabil Ahmed, Xiaoliu Zhang

**Affiliations:** 1 Department of Biology and Biochemistry, University of Houston, Houston, Texas, United States of America; 2 Department of Molecular Virology and Microbiology, Baylor College of Medicine, Houston, Texas, United States of America; 3 Center for Cell and Gene Therapy, Baylor College of Medicine, Houston, Texas, United States of America; New York University, United States of America

## Abstract

A simple and sensitive method to quantitatively measure the cytolytic effect of tumor-specific T killer cells is highly desirable for basic and clinical studies. Chromium (^51^Cr) release assay has been the “gold standard” for quantifying cytolytic activities of cytotoxic T lymphocytes (CTLs) against target cells and this method is still being used in many laboratories. However, a major drawback of this method is the use of radioactive materials, which is inconvenient to handle because of environmental safety concerns and expensive due to the short half-life of the isotope. Consequently, several nonradioactive methods have been reported recently. Here we report a new method that we recently developed for quantifying antigen-specific cytolytic activity of CTLs. This method fully exploits the high sensitivity and the relative simplicity of luciferase quantitative assay. We initially expected the released luciferase in the supernatant to be the adequate source for monitoring cell death. However, to our total surprise, incubation of these killer T cells with the tumor cell targets did not result in significant release of luciferase in the culture medium. Instead, we found that the remaining luciferase inside the cells could accurately reflect the overall cell viability.

## Introduction

Cytotoxic T lymphocytes (CTLs) play an important role in the host's defense against intracellular pathogens and malignant cells [1]. A simple and sensitive method to measure their activity would greatly benefit basic and clinical studies. For a long time, chromium (^51^Cr) release assay has remained as the “gold standard” for quantifying cytolytic activities of CTLs against target cells and this method is still being used in many laboratories around the world [2], [3]. However, a major drawback of the ^51^Cr release assay is the use of radioactive materials, which are inconvenient to handle because of environmental safety concerns and expensive due to the short half-life of the isotope. Consequently, several nonradioactive methods have been reported recently. One such method measures the release of endogenous enzymes (e.g., lactate dehydrogenase) in the supernatant during the CTL-mediated cytolysis of target cells [4]. However, dead effector cells could also release the same enzyme, which may compromise the accuracy of quantification by this method. Another method, reported recently, uses fluorescent dye to label the target cells [5] or transduces target cells with the gene encoding the green fluorescent protein [6], [7]. However, disadvantages of these methods include high spontaneous release of the fluorescent dye, low intensity of the fluorescence signal, and the requirement of expensive and sophisticated equipments such as flow cytometry.

Here we report a new method that we recently developed for quantifying antigen-specific cytolytic activity of CTLs. This method fully exploits the high sensitivity and the relative simplicity of luciferase quantitative assay, while avoiding the disadvantages of radioactive methods. We initially transduced target cells with a piggyBac transposon/transposase vector containing a fusion gene of GFP and luciferase, by which stable cell lines containing the fusion gene could be conveniently selected and established. We then examined the feasibility of using quantitative assays of luciferase activity to determine the cytolytic effect of modified T cells that can specifically recognize these tumor cells. We initially expected the released luciferase in the supernatant to be the adequate source for monitoring cell death. However, to our total surprise, incubation of these killer T cells with the tumor cell targets did not result in any detectable luciferase activity in the culture medium. Instead, we found that the remaining luciferase inside the cells could accurately reflect the overall cell viability.

## Materials and Methods

### Plasmid construction

Construction of pIR-Her2 plasmid. The HER2 sequence was cut out from a HER2 WT plasmid, which was kindly provided by Dr. Mien-Chie Hung (M.D. Anderson Cancer Center). The entire gene was cleaved out using HindIII and blunt-end ligated into the piggyBac-containing plasmid pIR-eGFP [8], which had been digested with XhoI and NotI. This replaced the GFP gene in pIR-eGFP with HER2. The new plasmid is designated pIR-Her2.

Construction of pIR-GFP-luc plasmid. The GFP and luciferase fusion gene, eGFP-luc, was cut out from the SFP-eGFP-luc plasmid with XbaI and MluI. Then eGFP-luc was blunt-end ligated into pIR-eFGFP which had been digested with BamHI. This replaced the GFP gene in pIR-eGFP with the eGFP-luc fusion gene. The new plasmid was designated pIR-eGFP-luc.

### Establishment of a stable tumor cell line expressing both HER2 and eGFP-luc

4T1 cells are a 6-thioguanine-resistant cell line derived originally from a BALB/c spontaneous mammary carcinoma [9] and was kindly provided by Dr. Fred Miller (Michigan Cancer Foundation, Detroit, MI, USA). Initially 4T-1 cells were co-transfected with three plasmids: pIR-Her2, pIR-eGFP-luc and pCMV-piggyBac. pCMV-piggyBac contains the piggyBac transposase that will recognize the ITR sequence in the other two plasmids and enforce integration [10]. After transfection, the cells were selected with puromycin at a concentration of 2 µg/ml. Then GFP-positive cells were sorted to more than 90% purity with BD FACS AriaII (BD Biosciences, San Jose, California). The sorted GFP positive cells were then seeded as single cells in 96-well plate by limiting dilution and screened for colonies expressing HER2 by staining them with PE conjugated -anti-human HER2 antibody (BioLegend, San Diego, CA).

### Transduction of murine splenocytes with retroviral vector containing chimeric TCR

The construction of the retroviral vector (SFG-Her2-CD28-Zeta) containing chimeric TCR that specifically recognizes HER2 has been described [11]. To produce the retroviral vector stock, SFG-Her2-CD28-Zeta were co-transfected with the packaging plasmid, pCL-ECO, by using lipofectamine 2000 (Invitrogen, Carlsbad, CA). Supernatants containing the retrovirus were collected 48 and 72 h later and were used to transduce freshly harvested splenocytes from Balb/C mice. Briefly, splenocytes were harvested from Balb/C mice and cultured with RPMI 1640 medium supplemented with 25 mM HEPES, 200 nM L-glutamine, 10% FBS, 1% MEM non-essential amino acids, 1 mM sodium pyruvate, 50 µM β-mercaptoethanol, 100 µg/ml streptomycin and 100 U/ml penicillin. Single-cell suspensions (2×106/ml) were activated with Concanavalin A (2 µg/ml; Sigma, St. Louis, MO) for 24 h. The stimulated cells were transduced with Her2-CD28-Zeta retrovirus supernatant in the RetroNectin (Takara Bio. Inc., Shiga, Japan) precoated non–tissue culture 24-well plate. The retroviral vector routinely transduces the cells at approximately 50% efficiency. The transduced splenocytes were then cultured for 48 hours in fresh medium supplemented with mIL-2 (10ng/ml) to allow the cells to recover. They were then used directly for T cell mediated cytotoxicity assay.

### Quantification of cell viability and luciferase activity

The cell viability was determined by Trypan blue exclusion assay after counting viable cells with a hemocytometer. All luciferase assays were performed with Bright-Glo™ Luciferase Assay System (Promega, Madison, WI), and were conducted essentially according to the manufacturer's protocol. For measuring cell-associated luciferase activity, culturing medium was removed. The mixture of 50 µl of the prepared luciferase reagent and 50 µl PBS was added to each well of the 96-well plates and the plates were shaken gently for 5 minutes for the cells to be completely lysed. The lysed mixture was then transferred into the provided plate for luminescence measurements with a luminometer. For measuring luciferase activity in the medium, 50 µl medium was directly mixed with 50 µl of the prepared luciferase reagent. Luminescence measurements were performed with a SpectraMax® multi-mode microplate reader (Molecular Devices, Sunnyvale, CA).

### Statistical analysis

All data were expressed as mean ± standard deviation. Correlation coefficient analysis was performed with Pearson's product-moment coefficient method.

## Results

### Establishment of stable tumor cell lines that express firefly luciferase

To facilitate the establishment of the stable cell lines, we employed the piggyBac transposon/transposase vector system. This system requires only the inverted terminal repeats (ITRs) to flank a transgene and transient expression of the transposase enzyme to catalyze the gene insertion into the chromosome for stable gene expression [12]. This vector system has also been shown to be able to insert multiple genes simultaneously [10]. For this study, we constructed two ITR containing vectors. The first one is pIR-gfp-luc, which contains a fusion gene of *GFP* and firefly *luciferase*. We also constructed a transposon-based vector that contains the *HER2* gene flanked by ITR (pIR-Her2). The gene product, human epidermal growth factor receptor 2 (HER2), serves as a tumor antigenic target for T cell recognition and killing. These two plasmids, together with a plasmid containing *transposase* gene (pCMV-piggyBac) were co-transfected into a murine mammary tumor cell line, 4T1, which is a well-established syngeneic murine tumor cell line that can naturally metastasize to many organ tissues [9]. Cells were initially selected for stable GFP expression and cloned. Individual clones were then screened for HER2 expression by flow cytometry. The majority of the GFP-positive cell clones were found to also be positive for HER2 expression. One of the clones that expresses both GFP and HER2, designated 4T1-Her2, was chosen for the subsequent studies.

**Figure 1:**
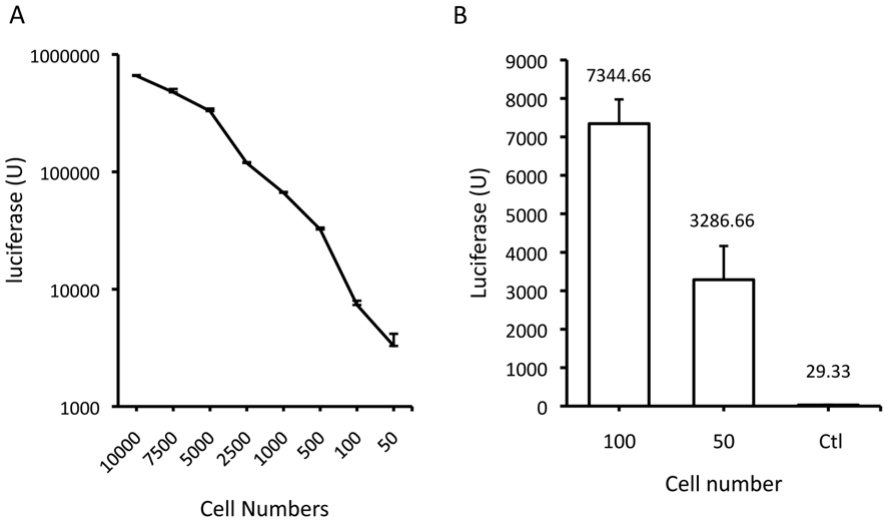
Quantification of luciferase expression from serially diluted 4T1-Her2 cells. **A**. Cells were seeded at a large range of density (from 10,000 to 50 per well) and were assayed for luciferase expression 2 h later. **B**. The assay was focused on the low cell number wells. The numbers above each bar indicate the actual luciferase units detected.

To determine luciferase activity from the established cell line, we seeded 4T1-Her2 cells in triplicate into 96-well plates at different numbers, starting from ten thousand cells per well, gradually decreasing to 50 cells per well. Cells were lysed two hours after seeding for quantification of luciferase activity. The results showed that there was good correlation between the level of luciferase activity and the number of cells seeded (Fig. 1A). Moreover, the data confirm that the luciferase assay is indeed extremely sensitive. A luciferase activity of more than 3,000 units was detected from a mere 50 cells against a barely detectable background (Fig. 1B).

### High correlation coefficiency between target cell numbers and luciferase activity

**Figure 2:**
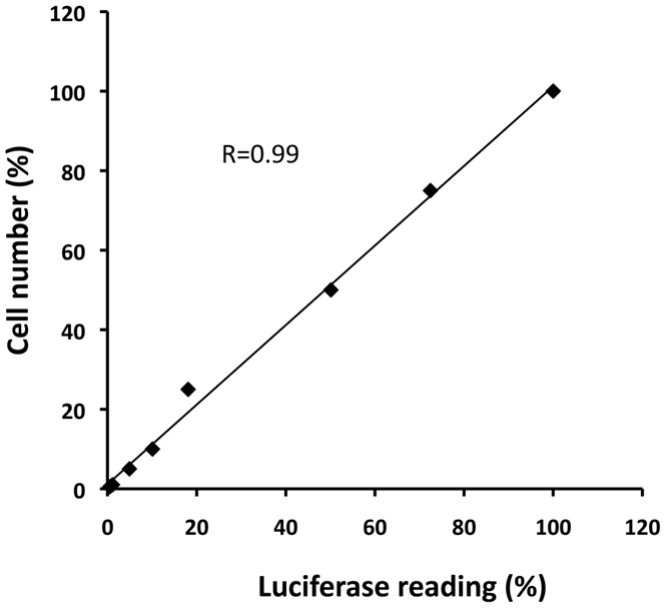
Pearson's correlation coefficient analysis. 4T1-Her2 cells were seeded in 96 well plates at increasing numbers and the luciferase level was determined after 2 hours. Both cell number and luciferase quantity were converted into percentage by dividing the individual figure with the maximal cell number seeded (100,000) or the luciferase reading from the well with the maximal cell number. The correlation coefficient (R) is indicated in the figure.

To be a sensitive and reliable quantification method, a high correlation coefficiency between the sample quantity and the readout is crucial. Thus, we calculated the coefficiency from an experiment in that different numbers of cells seeded in 96-well plates were measured for luciferase activity. The cell numbers were converted into percentage by dividing them with the maximal number of cells used in this experiment, as was the luciferase activity from each group by dividing the reading with the reading from the maximal cell number group. Then the data was plotted. Pearson analysis showed that there is an extremely good correlation between the cell numbers and the level of luciferase activity (R = 0.99, Fig. 2), indicating that this detection method could accurately measure the cell viability in a large range of cell numbers.

### Luciferase does not leak to the supernatant even at the peak of CTL-mediated cytolysis

**Figure 3:**
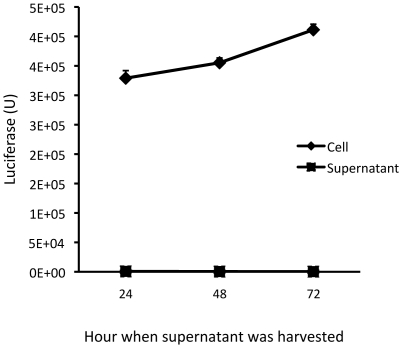
Low natural release of luciferase from 4T1-Her2 cells. Initially 10,000 4T1-Her2 cells were seeded. The supernatants were collected at the indicated times for measurement of luciferase.

One of the main drawbacks with the fluorescent dye labeling approach is the non-specific release of the labeling agent, which detrimentally affects the detection specificity. To examine the level of natural release of luciferase from the established cell line, we seeded 10,000 4T1-Her2 cells into a 96-well plate. The supernatants were collected at different times for quantification of luciferase activity. The results show that the non-specific release of luciferase in the supernatant was barely detectable even in wells where the cell-associated luciferase activity was detected at an extremely high level (over 400,000 units, Fig. 3), indicating that non-specific release is not an issue with this detection method.

**Figure 4:**
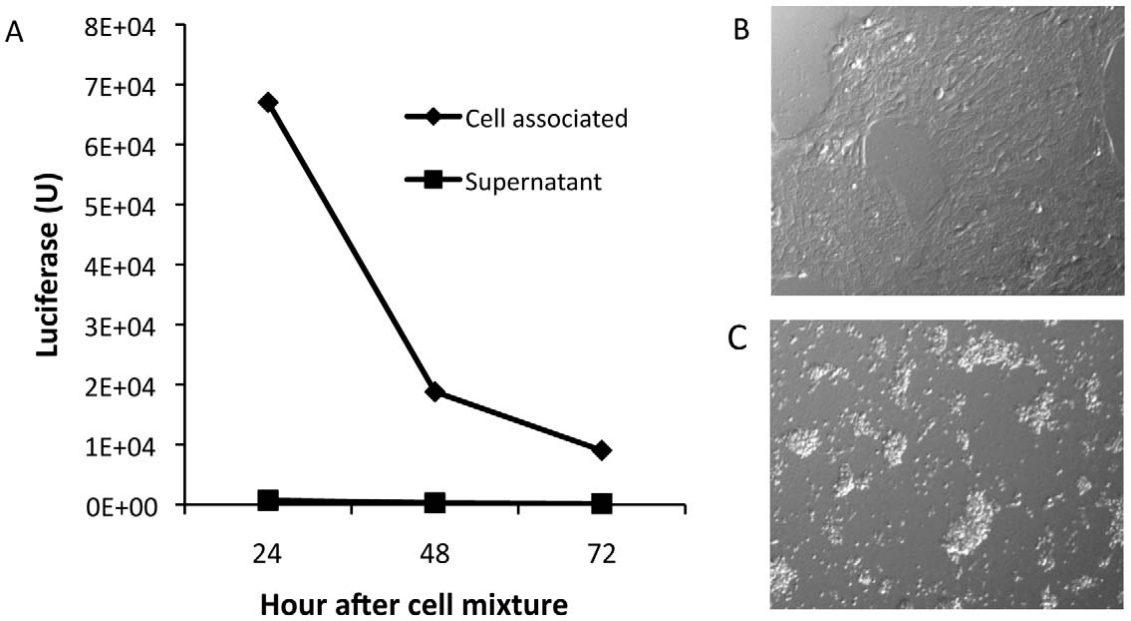
Luciferase was undetectable in the supernatant during T cell-mediated 4T1-Her2 cytolysis. **A**. Cell-associated and supernatant luciferase activity during cTCR-splenocyte mediated cytolysis of 4T1-Her2 cells. cTCR-splenocytes were added to 4T1-Her2 cells at a ratio of 10∶1. Supernatants and cells were harvested at the indicated time for quantification of luciferase activity. **B**. 4T1-Her2 monolayer without cTCR-transduced splenocytes. **C**. Seventy-two h after cTCR-tranduced splenocytes were added to 4T1-Her2 monolayer.

Due to the extremely low non-specific release of luciferase in the supernatant, we considered that any luciferase that was released during CTL-mediated cytolysis would be detectable and thus would represent an extremely sensitive quantification method. For generating tumor-specific CTLs, we adopted a previous reported method in which a chimeric T cell receptor (TCR) was engrafted into naïve T cells to convert them into tumor-specific killer cells [11]. In this method, a single chain antibody specific to HER2 was fused to the external portion of the zeta chain of the TCR complex. Expression of the chimeric TCR enables T cells to recognize tumor cells expressing HER2 with pre-defined specificity and in a non-MHC restricted manner. We mixed the chimeric TCR-engrafted (cTCR) splenocytes with 4T1-Her2 cells at a 10∶1 ratio. Luciferase activity was determined from both supernatant and cell fractions. As the incubation progressed, cell-associated luciferase activity decreased steadily. However, the luciferase activity in the supernatant remained at a very low level even at 72 h after the cell mixture was set up (Fig. 4A), a time when severe cytopathic effect was clearly visible and the entire 4T1-Her2 monolayer was almost completely destroyed (Fig. 4C). These results indicate that during the cytolysis process, luciferase does not readily release to the outside and thus the supernatant is not the ideal source for monitoring cell viability based on luciferase assay.

### Quantification of decrease in cell-associated luciferase represents a reliable way of deducing cytolytic activity of killer T cells

**Figure 5:**
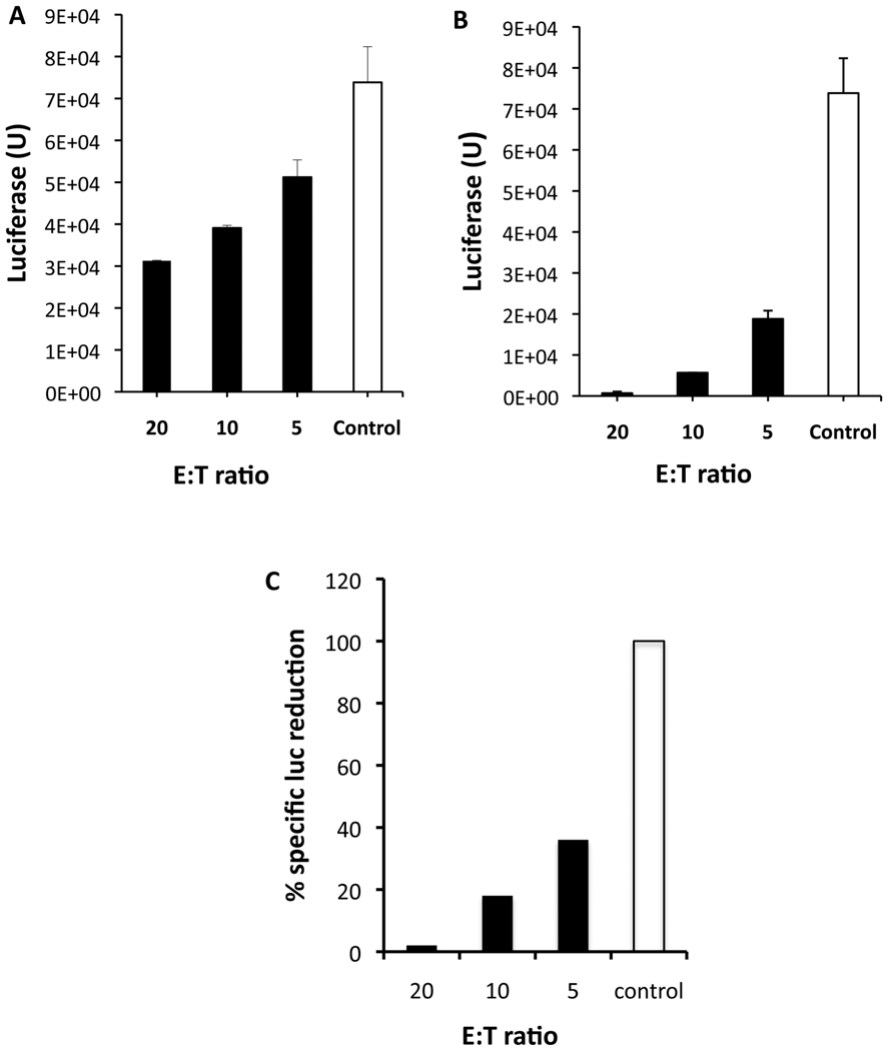
Reduction in cell-associated luciferase activity closely reflects the killing activity of T killer cells against tumor targets. Mock-transduced (**A**) or cTCR-transduced (**B**) splenocytes were added to 4T1-Her2 cells at the indicated ratio. Cells were harvested 72 later for quantification of luciferase activity. **C**. Using the formula: % specific luc reduction = (% luc reduction from engrafted T-cell)/(% luc reduction from control T-cell)×100, the data from **A** and **B** were converted into percentage of specific luciferase release and plotted.

The results in Fig. 4A show that, despite the absence of luciferase activity in the supernatant, the cell-associated luciferase activity was proportionally reduced during the T cell-mediated killing of 4T1-Her2 cells. Thus, we did another experiment to thoroughly monitor the luciferase activity inside the cells during the cytolysis of 4T1-Her2 cells by cTCR-transduced splenocytes. We mixed the effector cells with the target cells at different ratios and measured the luciferase activity from the cell mixture at time points from 24 to 72 h. We did not choose a shorter time point for this assay as the complete turn-over of luciferase, which has a half-life of 3 h when inside cells, will take approximately 12–15 h. Mock-engrafted splenocytes were used as a control to confirm the tumor-killing specificity of cTCR-transduced splenocytes. While the mock-treated splenocytes only marginally reduced the cell-associated luciferase activity (by approximately two-fold, Fig. 5A), incubation with cTCR-transduced splenocytes resulted in a dramatic reduction of luciferase activity in the target cells (Fig. 5B). When the effector cells were mixed with the target cells at a 10∶1 ratio, there was a 15-fold reduction of luciferase activity as compared with the control. When the ratio was increased to 20∶1, the luciferase activity was reduced by more than 100 fold, to a barely detectable level (Fig. 5C). Together these results show that cTCR T cells can efficiently and specifically lyse these tumor target cells and that detection of cell associated luciferase activity can be used to quantitatively monitor the lytic activity of these engrafted T killer cells.

## Discussion

Despite the proven reliability and sensitivity of the ^51^Cr release assay in measuring the cytolytic activities of CTLs, concerns related to handling radioactive materials have led to efforts to develop non-radioactive methods. Among all the recently reported new methods, fluorescence-based assays are the most promising. However, these methods also share one of the drawbacks associated with the old method; nonspecific release of the fluorescent labeling agent. This is particularly problematic in experiments where prolonged incubation of the effector and target cells is required. In this study, we show that firefly luciferase, once transduced into target cells, can serve as an attractive marker for measuring T cell cytotoxicity. This method has several unique features. First, luciferase has a relatively short half-life, estimated to be around 3 h after its synthesis inside the cells [13]. Thus, its quantitative fluctuation can accurately reflect the cell viability. Second, unlike other methods, its non-specific release is extremely low. This method is also highly sensitive, and recently developed detection kits (e.g., the Bright-Glo™ Luciferase Assay System) with a luminescence (signal) half-life around 30 min, have further increased the detection sensitivity. Finally, this assay is easy to perform and does not require expensive and sophisticated equipment.

Initially we set up the experiment to monitor the luciferase activity in the supernatant, as in other methods. Surprisingly, however, luciferase activity was virtually undetectable even when the severe cytopathic effect on target cells was clearly visible under the microscope. One explanation for this apparent discrepancy is probably due to the ultra short half-life of luciferase in the supernatant. It has been reported that half-life of firefly luciferase becomes even shorter (estimated to be 20–30 min) once it is released to the outside environment of cell [13]. As such, the released luciferase might have quickly gotten degraded. Another possibility is that, due to its relatively large mass (as compared with ^51^Cr or fluorochrome), it may not freely leak into the supernatant even though plenty of pores are punched on the target cell membranes by the perforins released from the effector cells. It has been reported that the size of the pores punctured by perforins are relatively small (around 16 nM in diameter), which may not allow protein molecules to freely pass through [14]. Regardless, our data show that measurement of cell-associated luciferase activity can be used to quantitate the cytolytic activity during T killer cell mediated oncolysis.

Although we did not directly compare our method with the “gold standard” ^51^Cr release assay, we believe that the sensitivity of these two methods is comparable. ^51^Cr release assay has been frequently used by many laboratories to measure the killing activity of T cells engrafted with chimeric TCRs [15], [16]. The detection sensitivity seems to be similar to the method described in this report when the amount of target cells and the E∶T ratios have been taken into consideration. The fact that luciferase from as little as 50 cells could be readily detected by our assay supports this assumption. A related issue is if this method is applicable to measure cytolytic activities from CTLs that are endogenously generated during natural antigen encounter or vaccination. Considering that our method has similar detection sensitivity as the widely used traditional ^51^Cr release assay, we feel confident that this method can be adapted for that purpose.
